# Characterization of carotenoids in *Rhodothermus marinus*

**DOI:** 10.1002/mbo3.536

**Published:** 2017-10-17

**Authors:** Emanuel Y. C. Ron, Merichel Plaza, Thordis Kristjansdottir, Roya R. R. Sardari, Snaedis H. Bjornsdottir, Steinn Gudmundsson, Gudmundur Oli Hreggvidsson, Charlotta Turner, Ed W. J. van Niel, Eva Nordberg‐Karlsson

**Affiliations:** ^1^ Division of Biotechnology Department of Chemistry Lund University Lund Sweden; ^2^ Division of Centre for Analysis and Synthesis Department of Chemistry Lund University Lund Sweden; ^3^ Matís ohf Reykjavik Iceland; ^4^ Center for Systems Biology University of Iceland Reykjavik Iceland; ^5^ Division of Applied Microbiology Department of Chemistry Lund University Lund Sweden

**Keywords:** Antioxidant, carotenoids, *Rhodothermus marinus*, salinixanthin

## Abstract

*Rhodothermus marinus,* a marine aerobic thermophile, was first isolated from an intertidal hot spring in Iceland. In recent years, the *R. marinus* strain PRI 493 has been genetically modified, which opens up possibilities for targeted metabolic engineering of the species, such as of the carotenoid biosynthetic pathway. In this study, the carotenoids of the *R. marinus* type‐strain DSM 4252^T^, strain DSM 4253, and strain PRI 493 were characterized. Bioreactor cultivations were used for pressurized liquid extraction and analyzed by ultra‐high performance supercritical fluid chromatography with diode array and quadropole time‐of‐flight mass spectrometry detection (UHPSFC‐DAD‐QTOF/MS). Salinixanthin, a carotenoid originally found in *Salinibacter ruber* and previously detected in strain DSM 4253, was identified in all three *R. marinus* strains, both in the hydroxylated and nonhydroxylated form. Furthermore, an additional and structurally distinct carotenoid was detected in the three strains. MS/MS fragmentation implied that the mass difference between salinixanthin and the novel carotenoid structure corresponded to the absence of a 4‐keto group on the ß‐ionone ring. The study confirmed the lack of carotenoids for the strain SB‐71 (*ΔtrpBΔpurAcrtBI’::trpB*) in which genes encoding two enzymes of the proposed pathway are partially deleted. Moreover, antioxidant capacity was detected in extracts of all the examined *R. marinus* strains and found to be 2–4 times lower for the knock‐out strain SB‐71. A gene cluster with 11 genes in two operons in the *R. marinus* DSM 4252^T^ genome was identified and analyzed, in which several genes were matched with carotenoid biosynthetic pathway genes in other organisms.

## 1 INTRODUCTION

*Rhodothermus marinus* is an aerobic heterotrophic gram‐negative bacterium that was first isolated from an intertidal hot spring at *Ísafjardardjup* in Iceland. This thermophilic bacterium has since been isolated from several marine environments (Bjornsdottir et al., 2006). The genus *Rhodothermus* is part of the family *Rhodothermaceae,* which has recently been replaced under *Bacteroidetes, Cytophagia*,* Incertae sedis II* (Ludwig, Euzéby, & Whitman, 2015). *R. marinus* first gathered interest due to its broad spectrum of thermostable carbohydrate degrading enzymes. These abilities make *R. marinus* a candidate for use in the bioconversion of renewable raw materials into high‐value chemicals.

*R. marinus* has recently been a target for genetic engineering, enabling gene knock‐out and the introduction of heterologous genes (Bjornsdottir, Fridjonsson, Kristjansson, & Eggertsson, 2007). This gives way for further opportunities, including genetic engineering for the production of carotenoid derivatives. (Bjornsdottir, Fridjonsson, Hreggvidsson, & Eggertsson, 2011) The possibility of production of natural or altered carotenoids highlights the importance of the understanding of the carotenoid biosynthetic pathway. A few genes of the pathway have previously been determined through knock‐out trials in *R. marinus* strain SB‐71. Strain SB‐71 (*ΔtrpBΔpurAcrtBI’::trpB*) has two carotenoid biosynthetic genes (*crtB* and *crtI)* knocked out, which normally catalyze two units of geranylgeranyl pyrophosphate to phytoene and phytoene to lycopene, respectively (Bjornsdottir et al., 2011). These mutations resulted in white colonies in contrast to its natural red color due to the absence of carotenoids.

Carotenoids and carotenoid glycosides are of interest due to their use as coloring agents in foods and chemical pre‐cursors to fragrances and flavor industries (Dembitsky, 2005). It has also been shown that consumption of vegetables with carotenoids such as ß‐carotene has health‐beneficial properties due to the conversion into vitamin‐A. *R. marinus* strain DSM 4253 and the heterotrophic *Salinibacter ruber,* both members of the *Rhodothermaceae* family, have been shown to produce monocyclic carotenoid glucoside esters with fatty acid residues, that is, salinixanthin (Lutnaes, Oren, & Liaaen‐Jensen, 2002; Lutnaes, Strand, Petursdottir, & Liaaen‐Jensen, 2004). In *S. ruber,* salinixanthin has been discovered to be a functional group in xanthorhodopsin, a protein/carotenoid complex that makes up a light‐driven proton pump. (Balashov et al., 2005).

Even though *R. marinus* type‐strain DSM 4252^T^ is the most studied of the strains in the genus, there are no reports on its carotenoid content. Moreover, characterization of the natural carotenoids and the carotenoid biosynthetic pathway of PRI 493 are required for the continuation of the genetic engineering and thus the production of carotenoid derivatives in *R. marinus*.

The aim of this study is to analyze and compare the carotenoids present in the aforementioned *R. marinus* strains and comparing these to the previously characterized monocyclic carotenoid glucoside ester, found in strain DSM 4253. In addition, the antioxidant capacity of the cell extracts is determined and correlated with the carotenoid biosynthetic pathway of *R. marinus*, which is proposed in this paper.

## 2 MATERIALS AND METHODS

### 2.1 Chemicals and reagents

All the chemicals were of analytical grade. 2,2′‐azinobis (3‐ethylbenzothiazoline‐6‐sulfonic acid) (ABTS) was purchased from Fluka (Buchs, Switzerland). 6‐Hydroxy‐2,5,7,8‐tetramethylchroma‐2‐carboxylic acid (Trolox), 2,2‐diphenyl‐1‐picrylhydrazyl, ammonium formate, and potassium persulfate were supplied by Sigma Aldrich (Steinheim, Germany). Formic acid was from Merck (Darmstadt, Germany). Methanol, LC‐MS grade, was provided by Scharlau (Barcelona, Madrid). Ultrapure Milli‐Q water was used (Millipore, Billerica, MA).

### 2.2 Cultivation and preparation of crude extracts

*Rhodothermus marinus* strains DSM 4252^T^ (R‐10) and DSM 4253 (R‐18) were obtained from the German Collection of Microorganisms and Cell Cultures (DSMZ). *R. marinus* strains PRI 493 and SB‐71 (*ΔtrpBΔpurAcrtBI’::trpB*) were received as freeze‐dried samples from Matís ohf, Reykjavík, Iceland. The strains were activated by streaking on solid agar with modified *Thermus* medium 162 with 1% NaCl at 65°C for 24–48 hr (Degryse, Glansdorff, & Pierard, 1978). Colonies were transferred into 5 mL liquid Lysogeny Broth (LB), 10 g/L tryptone (Duchefa Biochemie), 10 g/L NaCl, and 5 g/L yeast extract (Duchefa Biochemie), in two 15 ml falcon tubes and incubated in a shaking incubator at 65°C, 200 rpm for 24 hr. LB medium was used for all further cultivations. The strains were grown in 50 mL for 8 hours before inoculating (10% v/v) a bioreactor (Multifors 2, Infors). The bioreactor had a total working volume of 0.5 L and parameters were set to: 65°C, 1 vvm aeration, and pH 7. The stirrer speed was cascaded with a pO_2_ level at 40%. Growth was monitored through offline OD measurements at 620 nm. At the end of the exponential phase, the cells were harvested by centrifugation (Thermo Scientific Sorvall Lynx 4000 centrifuge) at 5500 g for 15 min at 10°C. The pellets were washed with a 0.9% (w/v) NaCl solution three times at the original liquid volume before freezing and subsequent freeze‐drying (LABCONCO, freeze dry system).

### 2.3 Pressurized liquid extraction

Extractions of *R. marinus* samples were performed using an accelerated solvent extractor (ASE 350, Dionex, Sunnyvale, CA), equipped with a solvent controller. To avoid any possible oxidation effects and to remove the dissolved oxygen, ethanol was sonicated for 45 min prior to use. Extractions were performed at 100°C for three extraction cycles (2 min per extraction cycle) based on an experimental method used for the extraction of carotenoids from microalgae (Plaza et al., 2012) with some modifications. Each extraction started with heating the extraction cell for 5 min. Extractions were performed in 10–11 ml extraction cells, containing 0.2–0.6 g of freeze‐dried sample. The extraction procedure was as follows: (1) the extraction cell was loaded into the oven; (2) the cell was filled with solvent up to a pressure of 1500 psi; (3) heat‐up time was applied, (4) a static extraction was performed with all system valves closed; (5) the cell was rinsed (with 60% cell volume using extraction solvent); (6) solvent was purged from the cell with N_2_ gas and (7) depressurization took place. Between extractions, the complete system was rinsed to prevent extract carry‐over. The extracts obtained were dried with a Reacti‐Vap^TM^ Evaporator (Thermo Fisher, Germering, Germany) until total sample dryness. The dry extracts were redissolved in ethanol at 10 mg/ml concentration and filtered through 0.2 μm PTFE filters (VWR International, West Chester, PA) and were ready for UHPSFC separation without further clean‐up. The extracts obtained were stored at −80°C.

### 2.4 Analysis of carotenoids by UHPSFC‐DAD‐QTOF/MS

The analysis of carotenoids was performed using a Waters Acquity Ultra Performance Convergence Chromatography (UPC^2^) system (Waters, Milford, MA) coupled to a photodiode array detector (PDA) and a quadrupole and orthogonal acceleration time‐of‐flight tandem mass spectrometer (XEVO‐G2 QTOF) with electrospray ionization (ESI) (Waters, MS Technologies, Manchester, UK). The system was controlled by Waters^®^ Empower^TM^ Chromatography software; while MassLynx^TM^ (V 4.1, SCN 779, Waters Corp., Manchester, UK) was used for MS data acquisition and treatment. Separation was carried out with CSH fluoro‐phenyl (100 mm × 3 mm, 1.8 μm) from Waters (Milford, MA). The mobile phase consisted of (A) CO_2_ and (B) methanol in a gradient elution analysis programmed as follows: 0 min, 5% (B); 0–1 min, 5% (B); 1–3 min, 5–15% (B); 3–7 min, 15% (B); 7–8 min, 15–5/% (B); and 8–10 min, 5% (B). The flow rate was 1.5 ml/min; injection volume, 5 μL; column temperature, 30°C, and backpressure, 160 bar. Ammonium formate (0.2% ) in methanol was used as a make‐up solvent, 0.5 ml/min. UV‐vis spectra were recorded in the range of 200–500 nm. The ESI interface was operated in positive mode, and full‐scan HPLC‐qTOF‐MS spectra were obtained by scanning from *m/z* 50 to *m/z* 1,000. The mass spectrometer was calibrated using a solution of sodium formate. Data were collected in continuum mode and mass was corrected during acquisition using an external reference (Lock‐Spray^TM^) comprising of 10 μL/min solution of leucine‐enkephalin (2 ng/μl) via a lock‐spray interface. The capillary and cone voltage were set at 3 kV and 40 V, respectively. Nitrogen was used as both cone gas (50 L/hr) and desolvation gas (800 L/h). The source and desolvation temperature were set at 150 and 300°C, respectively. Simultaneous acquisition of exact mass at high and low collision energy, MS^E^ (where E represents collision energy), was used to obtain full‐scan accurate mass fragment, precursor ion, and neutral loss information. The collision energy in function 1 (low energy) was off while in function 2 (high energy) and the collision energy ranged between 15 V to 60 V.

The MS/MS analyses were acquired by automatic fragmentation where the four different carotenoids found in the samples were fragmented. Data were collected in centroid mode. Collision energy values for MS/MS were adjusted as follows: *m/z* 940.64 and 924.64, 40 V; and *m/z* 926.64 and 910.65, 30 V. To clarify if the double ion peaks were from a single carotenoid in peak 3, a few drops of NaOH (0.5 mmol/L) was added to the make‐up solvent to increase Na^+^ adducts.

### 2.5 Trolox equivalent antioxidant capacity (TEAC) assay

The TEAC assay, based on a previously described method (Re et al., 1999) with some modifications, was used to measure the antioxidant capacity of the extracts. ABTS radical cation (ABTS^·+^) was produced by reacting 7 mmol/L ABTS with 2.45 mmol/L potassium persulfate and allowing the mixture to stand in the dark at room temperature for 12–16 hr before use. The aqueous ABTS^·+^ solution was diluted with ethanol to an absorbance of 0.70 (±0.02) at 734 nm. One milliliter of ABTS·^+^ radical solution was added to 10 µL of each sample concentration. After 50 min at room temperature, 300 μl of the mixture was transferred into a well of the microplate, and the absorbance was measured at 734 nm on a microplate spectrophotometer reader (Multiskan GO, Thermo Fisher, Germering, Germany). Trolox was used as a reference standard and results were expressed as TEAC values (mmol Trolox/g dry bacterial extract). These values were obtained from at least four different concentrations of each extract tested in the assay giving a linear response between 20% and 80% of the initial absorbance. All analyses were done at least in triplicate for each extract.

### 2.6 DPPH radical scavenging assay

The antioxidant capacity of all the obtained extracts was measured using the DPPH (2,2‐diphenyl‐1‐picrylhydrazyl) radical scavenging assay based on the protocol by Brand‐Williams et al. (1995). Briefly, a solution was prepared by dissolving 23.5 mg of DPPH in 100 ml of methanol. This stock solution was further diluted with methanol 1:10. Both solutions were stored at 4°C until use. Four different concentrations of extracts were tested. Twenty‐five microliter of these solutions were added to 975 μl of DPPH diluted solution to complete the final reaction medium (1 ml). After 4 hr at room temperature, 300 μl of the mixture was transferred into a microplate well and the absorbance was measured at 516 nm in a microplate spectrophotometer reader (Multiskan GO, Thermo Fisher). DPPH‐methanol solution was used as a reference sample. The DPPH concentration remaining in the reaction medium was calculated from a calibration curve. The percentage of remaining DPPH against the extract concentration was then plotted to obtain the amount of antioxidant necessary to decrease the initial DPPH concentration by 50% or EC_50_. Therefore, the lower the EC_50_, the higher the antioxidant activity. For rational reasons of clarity, the antioxidant capacity was determined as the inverse value of the efficient concentration EC_50_ (mg/ml), representing a comparable term for the effectiveness of antioxidant and radical scavenging capacity (1/EC_50_). The larger the antioxidant capacity, the more efficient an antioxidant. Measurements were done at least in triplicate for each extract.

## 3 RESULTS AND DISCUSSION

The *R. marinus* strains DSM 4252^T^, DSM 4253, and PRI 493 appeared as red colonies on medium 162, while strain SB‐71 (*ΔtrpBΔpurAcrtBI’::trpB*) produced white colonies. Low cell densities of *R. marinus* resulted in a long lag phase and a procedure for maintaining the exponential growth during transfers was therefore developed. In short, colonies were transferred into 5 mL liquid LB medium for precultivation and diluted 10‐fold into baffled shake flasks after 24 hr. The cells were then grown for 8 hr before inoculating the bioreactor, which were harvested at the end of the exponential phase.

The carotenoids of *R. marinus* were extracted using pressurized liquid extraction (PLE). The cell extract were red for all strains except for strain SB‐71 (*ΔtrpBΔpurAcrtBI’::trpB*), while the cell pellets were colorless (see supplementary data). Extraction conditions (solvent, temperature and time) were selected based on previous results obtained with the microalgae *Chlorella vulgaris* (Plaza et al., 2012). PLE uses solvents at elevated temperatures subjected to high enough pressures to keep the solvents in a liquid state during the extraction process. The extraction process is faster and more efficient at higher temperature due to faster diffusion rates (Björklund, Nilsson, & Bøwadt, 2000) and the high pressure that penetrates the sample matrix(Mustafa & Turner, 2011). In order to decrease the risk of carotenoid breakdown due to high temperature, a lower extraction temperature was used in comparison to literature and more importantly, a very short extraction time of 6 min was applied (Cha et al., 2010). Conventional carotenoid extraction procedures use organic solvents, harmful both for the environment and for human health, which has made PLE increasingly popular, especially for analytical purposes(Mustafa & Turner, 2011). Several studies have successfully used PLE for the extraction of carotenoids (Mustafa & Turner, 2011).

### 3.1 Analysis of carotenoids by UHPSFC‐DAD‐QTOF/MS

In order to obtain a suitable separation between the lipids and carotenoids of the bacterial crude sample, modification of the UHPSFC method was necessary. Hence, several columns were screened for optimal separation, including: Torus diethylamine (DEA), Torus high density diol (DIOL), Torus 1‐aminoanthracene (1‐AA), fluoro‐phenyl (CSH‐FP), and high strength silica C18 SB (HSS C18 SB). Only the CSH‐FP column resulted in acceptable separation of the carotenoid peaks and the rest of the lipids. The CSH‐FP column was selected and used throughout the analysis of the crude extracts and was further optimized by modification of gradient programs and flow rates (for best conditions, see material and methods).

**Figure 1:**
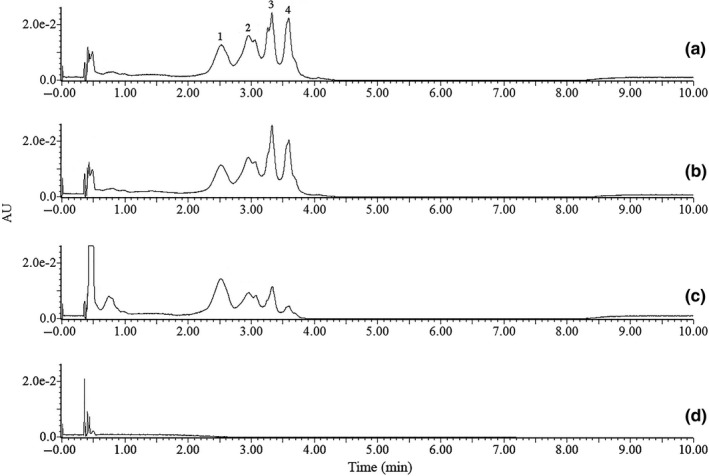
Chromatogram displaying the absorption at 450 nm for strain (a) DSM 4252^T^, (b) DSM 4253, (c) PRI 493, and (d) SB‐71. For peak identification, see Table 2

**Figure 2:**
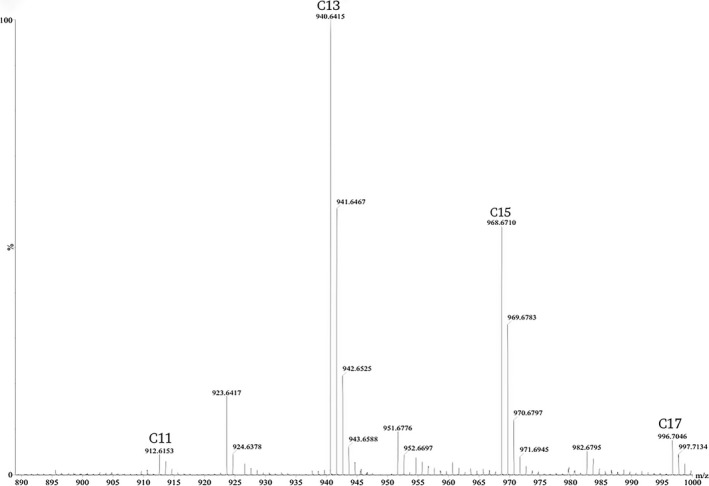
Spectrum of salinixanthin with variable fatty acid lengths (C_11_–C_17_) in *R. marinus* strain DSM 4252^T^ in peak 4 of the chromatogram

The chromatogram produced several peaks for *R. marinus* strains DSM 4252^T^, DSM 4253, and PRI 493 with retention times of 2.52, 2.96, 3.33, and 3.59 min, respectively (Figure 1). As expected, no absorption could be detected with the DAD for the *R. marinus* SB‐71 knock‐out strain, indicating the absence of carotenoids. The co‐eluting carotenoid peaks were determined to be chain length variations of the carotenoid acyl group, which was verified by MS analysis. The C_11_–C_17_ fatty acid pattern (Figure 2) could be detected in all of the chromatogram peaks and in *R. marinus* strains DSM 4252^T^, DSM 4253, and PRI 493. It has previously been reported that *R. marinus* DSM 4253 produces iso‐C_15_, anteiso‐C_15_, iso‐C_17_, and anteiso‐C_17_ fatty acids (Lutnaes et al., 2004) but that the composition greatly varies depending on the growth conditions including medium composition (Bjornsdottir et al., 2006). The carotenoids with iso13:0 fatty acid group consistently had the highest peak intensity in the MS, while the C_15_ fatty acid variant had the second highest intensity. Peak intensity of similar compounds with the same ionization characteristics can be assumed to have a proportional correlation with concentration, which would indicate a higher concentration of the carotenoid with the iso13:0 fatty acid constituent (Tang & Kebarle, 1993). The previously characterized hydroxylated salinixanthin (C_61_H_92_O_9_) (Lutnaes et al., 2002) was identified in peak 4 at the retention time of 3.60 min, having a mass of 968.677 *m/z*, Δ3 mDa relative to the theoretical mass. The identification of salinixanthin showed that the extraction method used was successful in retaining the carotenoids and that the selected chromatographic method was suitable for the separation. The MS results of the iso13:0 carotenoid variants in all four peaks of the *R. marinus* DSM 4252^T^ chromatogram are presented in Table 2.

In the third peak of the chromatogram, the carotenoids were found in both [M]^•+^ and [M+H]^+^ ionized forms at the masses of 952.6827 (Δ3.2 mDa, C_61_H_92_O_8_) and 953.6876 (Δ0.6mDa, C_61_H_93_O_8_), which were identified as the nonhydroxylated Salinixanthin with a iso15:0 fatty acid also reported by Lutnaes et al., (2004). Sodium was added in the mass spectrometry make‐up solution to exclude that the double ions peaks were two different compounds. The sodium addition resulted in peak intensity decrease for the [M]^•+^ and [M+H]^+^ peaks and a single new peak of 975.6691 *m/z* [M+Na]^+^ emerged, indicating that both [M]^•+^ and [M+H]^+^ ions originated from a single compound. It has previously been reported that structurally similar carotenoids can have two simultaneous ions when using electro‐spray (ES) as ionization method (Rivera, Christou, & Canela‐Garayoa, 2014). Salinixanthin with and without the hydroxyl group could be detected in all of the *R. marinus* strains DSM 4252^T^, DSM 4253, and PRI 493.

An ion with the *m/z* of 938.6956 (Δ4.4 mDa, C_61_H_94_O_7_) was detected in peak 1 of the chromatogram at the retention time of 2.52 min. The mass difference between the carotenoid in peak 3 and this ion corresponds to the absence of one oxygen and addition of two hydrogen atoms, which suggests that the carotenoid does not contain the keto group on the β‐ionone ring. To further support the suggested nonketo carotenoids in peak 1 and 2, MS/MS fragmentation of the compounds was performed. Fragmentation of carotenoids with a 4‐keto β‐ionone ring, such as cantaxanthin, forms a characteristic 203.1 *m/z* 4‐keto‐carotenoid fragment (Rivera et al., 2014). These fragments could be detected in peaks 3 and 4. However, such fragments could not be detected for the carotenoids in peak 1 and 2, which verifies that the mass difference is located on the β‐ionone ring.

**Figure 3:**
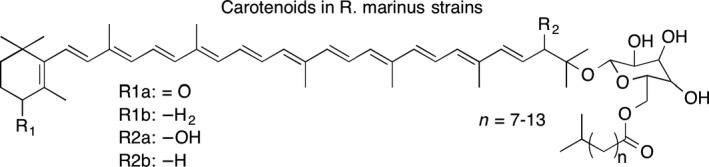
Proposed carotenoids in *R. marinus* strains DSM 4252^T^, DSM 4253, and PRI 493

A mass difference comparable to the hydroxylated and nonhydroxylated salinixanthin was found between peak 1 and 2. Hence, the ion 954.6934 *m/z* (Δ1.5 mDa, C_61_H_94_O_8_) in peak 2 corresponds to the hydroxylated form of the carotenoid in peak 1. Moreover, the same C_11_‐C_17_ fatty acid pattern of salinixanthin in peak 3 and 4 could also be seen in the carotenoids of peak 1 and 2. All of the four peaks contain typical MS/MS fragments originating from the polyene chain of carotenoids as well as dominant fragments at 341 and 359 *m/z*, which correspond to the masses of the acyl glucoside constituent, confirming overall structural similarity between the carotenoids in peaks 1–4. These carotenoids could be detected in *R. marinus* strains DSM 4252^T^, DSM 4253, and PRI 493 but not in SB‐71. Proposed structures of the carotenoids based on MS and MS/MS analyses can be seen in Figure 3.

Similar monocyclic carotenoid glycoside esters have previously been described in a few other microorganisms but not for species of the *Rhodothermaceae* family. Hydroxylated forms have been reported as phleixanthophyll in *Nocardia kirovani* (Guinand, Vacheron, & Michel, 1970) and nonhydroxylated in *Chloroflexus aurantiacus* (Takaichi, Tsuji, Matsuura, & Shimada, 1995) and in species of the *Myxococcus* genus (Dembitsky, 2005). The reason why the previous report on carotenoids in *R. marinus strain* DSM 4253 (Lutnaes et al., 2004) did not detect the carotenoids found in this study could be due to their loss in the purification steps that were performed in that study.

### 3.2 Bioinformatics analysis of putative carotenoid biosynthetic pathway genes

**Figure 4:**
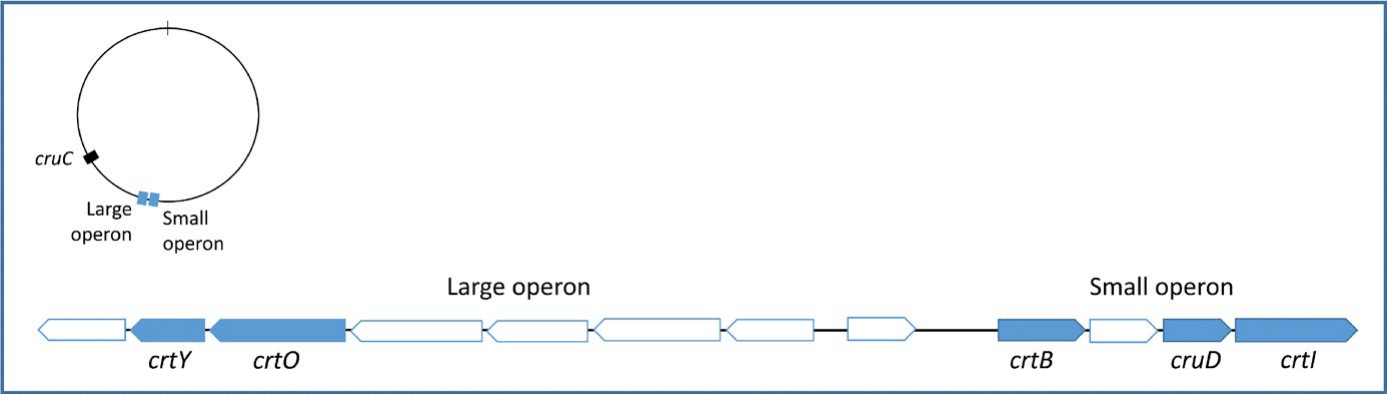
Putative carotenoid biosynthetic genes in *R. marinus* DSM 4252^T^. A genome map shows the relative location of the identified gene cluster and the nonclustered gene, *cruC*. The gene cluster, which contains two operons, is shown with the genes encoding proteins involved in the carotenoid biosynthetic pathway in blue

**Figure 5:**
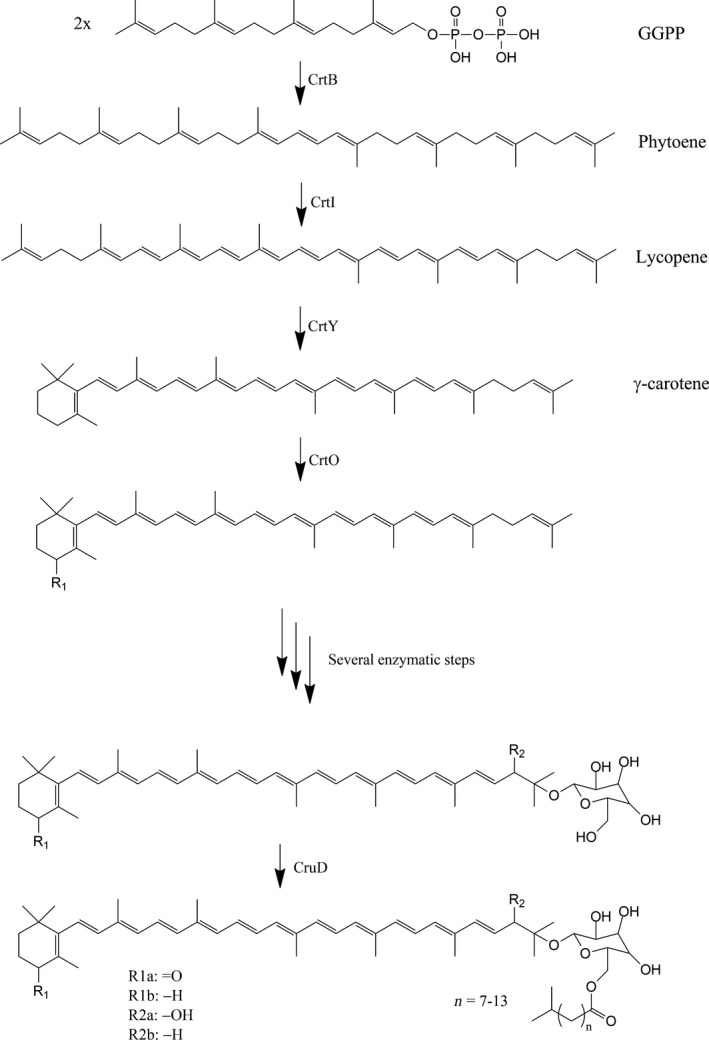
Putative carotenoid biosynthetic pathway gene cluster in *R. marinus* DSM 4252^T^ showing molecular structures and enzyme names. The steps are catalyzed by enzymes encoded by putative genes in the genome (CrtB, CrtI, CrtY, CrtO, and CruD)

A gene homology search for known carotenoid biosynthetic genes was performed for the *R. marinus* DSM 4252^T^ genome (Bjornsdottir et al., 2006). In this search, six genes encoding homologues to enzymes in carotenoid biosynthesis pathways from other species were found (CrtB, CrtI, CrtY, CrtO, CruD, and CruC), and allowed identification of a gene cluster, with two adjacent operons, one small and one larger (Figure 4). They appear to share a regulatory region, while being transcribed in opposite directions. The smaller operon consists of four genes and the larger of seven genes. A putative biosynthetic pathway for carotenoid production in *R. marinus* was suggested based on the results (Figure 5).

In the suggested carotenoid biosynthesis pathway (Figure 5), geranylgeranyl pyrophosphate is transformed into phytoene, catalyzed by phytoene synthase (CrtB) and subsequently converted to lycopene by phytoene desaturase (CrtI). The corresponding genes are found in the smaller operon of the cluster. Lycopene is further converted to γ‐carotene by lycopene cyclase (CrtY), which is encoded by a gene found in the larger operon. The salinixanthin of peaks 3 and 4 of the chromatogram had a keto group. A gene encoding a ketolase (CrtO), which converts γ‐carotene into 4‐keto‐γ‐carotene, is found in the larger operon (Figure 4).

In biosynthesis of structurally similar carotenes in other species (Richter, Hughes, & Moore, 2015; Takaichi et al., 2001) a 1′,2′‐hydratase (either CrtC or CruF, from different protein families) hydrates the double bond between C‐1′ and C‐2′. No homologues to these enzymes were, however, encoded by the genes in the *R. marinus* genome. The gene encoding 3′,4′‐desaturase (CrtD), a short chain dehydrogenase of the Rossmann fold, was also not unequivocally found in the *R. marinus* genome. Finally, part of the carotenoids undergo a 2′‐hydroxyl addition, and for this purpose spheroidene monooxygenases (CrtA) from *Rhodobacterium sphaeroides* and *Flavobacterium* sp. have been shown to act both as carotenoid 2‐ketolases and—hydroxylases (Lee, Holtzapple, & Schmidt‐Dannert, 2010; Rählert, Fraser, & Sandmann, 2009), but corresponding genes were not found in the *R. marinus* genome. Enzymes with the above functions, although with different sequence conservation, may however be encoded by the yet unidentified genes in the cluster (Figure 4).

A glycosyltransferase, (CruC) adds a unit of glucose to the carotenoids and an acetyltransferase (CruD) finally links a fatty acid to the glucose (Figure 5). A gene encoding a CruD homologue is found in the smaller operon, whereas a gene located far from the gene cluster showed relatively high homology to the gene encoding CruC in *S. ruber* (Query cover 90%, Ident 57%). In order to verify the predicted genes in the two operons, knock‐out mutations of the separate genes are vital for the validation of the suggested carotenoid biosynthetic pathway in *R. marinus*.

The biological function of the carotenoids produced by *R. marinus* is still under debate. It has previously been argued that carotenoids stabilize the cell membranes in thermophilic conditions but identical carotenoids have been found in the mesophilic *S. ruber* (Lutnaes et al., 2004). In *S. ruber,* salinixanthin has been shown to be a functional group in xanthorhodopsin, a protein/carotenoid complex that makes up a light‐driven proton pump. (Balashov et al., 2005). However, homology searches between rhodopsin genes in different *bacteroidetes* species and the *R. marinus* genome fail to detect any matches.

### 3.3 Total antioxidant capacity of the bacterial extracts

The antioxidant capacity of PLE extracts obtained from *R. marinus* strains DSM 4252^T^, DSM 4253, PRI 493, and SB‐71 were measured using two different *in vitro* assays, that is, TEAC and DPPH. The latter method provided the EC_50_ value, which indicates higher antioxidant capacity with lower EC_50_ values. These two methods aim to provide the antioxidant capacities against two different radicals and to gain insight into their mechanism of action (Table 1). All the extracts were able to neutralize ABTS˙^**+**^ and DPPH˙ radicals. The extracts from strains DSM 4252^T^, DSM 4253, and PRI 493 showed similar antioxidant capacities while those were significantly lower for strain SB‐71. The DPPH assay showed 3‐ to 3.7‐fold decrease in antioxidant capacity of *R. marinus* SB‐71 extract in comparison to the carotenoid‐producing strains, while the TEAC assay showed a 1.7 to 1.9‐fold decrease. The difference between *R. marinus* strain PRI 493 and SB‐71 is the knock‐out of several carotenoid pathway genes (Bjornsdottir et al., 2011) and the mutant could therefore be used as a negative control during the antioxidant capacity assays, meaning that any difference between these two strains in antioxidant capacity is due to the presence or absence of the carotenoid biosynthetic pathway (Bjornsdottir et al., 2011). To the best of our knowledge, antioxidant capacity has neither been studied for the carotenoids produced by *R. marinus* nor for the structurally similar carotenoids of *S. ruber*. Antioxidant capacity data was therefore compared to that of cyanobacteria *Spirulina platensis, Synechocystis sp,*. and *Phormidium sp*. The supercritical fluid extracts of *S. platensis* had EC_50_ values ranging from 66.6 to 204.5 μg/ml (DPPH assay), whereas PLE extracts obtained with ethanol had EC_50_ values ranging from 83 to 100 μg/ml (Table 1). The *R. marinus* cell extracts showed comparable, but slightly lower, antioxidant capacities to those of *S. platensis* using the DPPH assay. In conclusion, these data show that the antioxidant capacity is similar to that of the cyanobacterial species that were used for comparison. Also, that a disruption of the carotenoid biosynthesis pathway decreases the capacity of *R. marinus*. It should, however, be emphasized that the antioxidant capacity of a cell extract does not depict the antioxidant capacity of the carotenoids only, but is the sum of all anti‐oxidizing components in the cell extract matrix. To quantify the antioxidant capacity between the carotenoids found in *R. marinus* and other organisms, purification and quantification of the carotenoids is needed. The TEAC values obtained from PLE extracts of cyanobacteria *Synechocystis* sp. and *Phormidium* sp. were higher compared to the values obtained from the *R. marinus* extracts by a factor of 7‐10 (Fernández‐González, Sandmann, & Vioque, 1997; Plaza et al., 2010; Rodríguez‐Meizoso et al., 2008).

**Table 1:** TEAC and DPPH antioxidant capacity values obtained for the studied cell extracts after the extraction by PLE. All measurements were done at least in triplicate

Sample	DPPH(EC_50_ μg/ml) ± SD	TEAC	
		(μmol trolox/g of extract) ± SD	(μmol trolox/g cell dry weight) ± SD
DSM 4252^T^	223.1 ± 10.4	42.3 ± 3.7	7.49 ± 0.65
DSM 4253	228.5 ± 6.4	45.2 ± 1.3	9.90 ± 0.28
PRI 493	276.2 ± 3.4	40.8 ± 2.1	7.01 ± 0.36
SB71	821.0 ± 52.6	23.7 ± 1.5	4.48 ± 0.28
*Spirulina platensis* ‐ (SFE (Mendiola et al., 2005; Herrero et al., 2005; Plaza et al., 2010)‐ (PLE, ethanol, 115°C, 15 min))(Mendiola et al., 2005; Herrero et al., 2005; Plaza et al., 2010)	66.6–204.5 83–100	– –	–
*Synechocystis sp*. (PLE, ethanol, 100°C, 20 min)(Fernández‐González et al., 1997)	–	407 ± 8	–
*Phormidium sp*. (PLE, ethanol, 100°C, 20 min)(Rodríguez‐Meizoso et al., 2008)	–	318 ± 22	–

**Table 2:** Results of UHPSFC‐DAD‐QTOF/MS analysis of the crude extracts of R. marinus strain DSM 4252^T^

Peak	RT (min)	Measured (*m/z*)	Formula	Theoretical *m/z*	Error (mDa)	Fragments	Proposed compound
1	2.52	910.6675	C_59_H_90_O_7_	910.6687	−1.2	535.4, 341.2, 95.1, 69.1	Carotenoid acyl glycoside
2	3.02	926.6627	C_59_H_90_O_8_	926.6636	−0.9	550.4, 341.2, 95.1, 69.1	Carotenoid acyl glycoside (hydroxyl)
3	3.29	924.6503	C_59_H_88_O_8_	924.6479	2.4	415.3, 341.2, 203.1, 95.1, 69.1	Salinixanthin
4	3.65	940.6415	C_59_H_88_O_9_	940.6428	−1.3	413.3, 341.2, 203.1, 95.1, 69.1	Salinixanthin (hydroxyl)

## 4 CONCLUSIONS

In this paper, an UHPSFC‐DAD‐QTOF/MS method was developed that could detect the carotenoids of *R. marinus*. MS analysis of crude bacterial samples could confirm the presence of salinixanthin, not only in *R. marinus* DSM 4253 but also in extracts of the *R. marinus* type‐strain DSM 4252^T^ and strain PRI 493. Moreover, a novel glycoside carotenoid ester was detected in all of the carotenoid‐producing strains. This carotenoid was found in both hydroxylated and nonhydroxylated forms similarly to those of salinixanthin but without a keto group on the ß‐ionone ring. A C_11_‐C_17_ fatty acid pattern was detected in this study, which agrees to a previously characterized composition for *R. marinus* DSM 4253. Here, a carotenoid biosynthetic pathway is suggested for *R. marinus*, based on results from the mass spectrometry analysis as well as on gene homology searches and comparison with published pathways for other organisms. Further work is required to verify the predicted genes through knock‐out mutations in order to validate the suggested pathway. Antioxidant capacities of *R. marinus* extracts were also measured during this work, using two different antioxidant capacity assays, DPPH and TEAC. By comparing the results for strains DSM 4252^T^, DSM 4253, and PRI 493 with those for the knock‐out mutant SB‐71 (*ΔtrpBΔpurAcrtBI’::trpB*), it could be deduced that the carotenoid biosynthetic pathway contributes to the detected antioxidant capacity.

## CONFLICT OF INTEREST

None declared.
