# Supplementary material for: Characterization of carotenoids in Rhodothermus marinus
Source: Microbiologyopen. 2017 Oct 17;7(1):e00536. doi: 10.1002/mbo3.536 (PMC5822342; doi:10.1002/mbo3.536)
Supplement: Supplementary file 1 [file MBO3-7-na-s001.docx]

**SUPPLEMENTARY DATA**

Mass spectrum of *Rhodothermus marinus* strain 4253 peak 1-4, identical specta were found for all three carotenoid producing strains. None of these peaks were detected in the knock-out strain SB-71.

| Strain: | Fragmentation data | | | | | |
| --- | --- | --- | --- | --- | --- | --- |
| 4252 |  |  |  |  |  |  |
| Peak 1 |  |  |  |  |  |  |
| Source of fragment | Detected mass | Formula | Theoretical Mass | Delta | Height | Proposed Ion |
| Target | 910.6691 | C59H90O7 | 910.6687 | 0.4 | 3.12E+04 | [M]*+ |
| Carotenoid | 535.4304 | C40H55 | 535.4304 | 0 | 2.59E+04 | [M+H]+ |
| Glu+Fa13 | 359.2452 | C19H35O6 | 359.2434 | 1.8 | 5.94E+04 | [M+H]+ |
| Glu+Fa13 | 341.2322 | C19H33O5 | 341.2328 | -0.6 | 1.68E+05 | [M+H]+ |
|  |  |  |  |  |  |  |
| Peak 2 |  |  |  |  |  |  |
| Source of fragment | Detected mass | Formula | Theoretical Mass | Delta | Height | Proposed Ion |
| Target | 925.6593 | C59H89O8 | 925.6557 | 3.6 | 1.24E+03 | [M+H]+ |
| Carotenoid | 549.4099 | C40H53O | 549.4096 | 0.3 | 6.60E+03 | [M+H]+ |
| Glu+Fa13 | 359.2401 | C19H35O6 | 359.2434 | -3.3 | 6.08E+03 | [M+H]+ |
| Glu+Fa13 | 341.2317 | C19H33O5 | 341.2328 | -1.1 | 7.05E+04 | [M+H]+ |
| Ketoring | 203.1419 | C14H19O | 203.1436 | -1.7 | 6.42E+04 | [M+H]+ |
|  |  |  |  |  |  |  |
| Peak 3 |  |  |  |  |  |  |
| Source of fragment | Detected mass | Formula | Theoretical Mass | Delta | Height | Proposed Ion |
| Target | 926.6586 | C59H90O8 | 926.6636 | -5 | 2.59E+03 | [M]*+ |
| Carotenoid | 567.4209 | C40H55O2 | 567.4202 | 0.7 | 1.55E+03 | [M+H]+ |
| Carotenoid | 550.4155 | C40H54O | 550.4175 | -2 | 1.71E+03 | [M]*+ |
| Glu+Fa13 | 359.2452 | C19H35O6 | 359.2434 | -1.7 | 2.04E+04 | [M+H]+ |
| Glu+Fa13 | 341.2321 | C19H33O5 | 341.2328 | -0.7 | 5.44E+04 | [M+H]+ |
|  |  |  |  |  |  |  |
| Peak 4 |  |  |  |  |  |  |
| Source of fragment | Detected mass | Formula | Theoretical Mass | Delta | Height | Proposed Ion |
| Target | 940.6452 | C59H88O9 | 940.6428 | 2.4 | 2.92E+02 | [M]*+ |
| Carotenoid | 523.3588 | C37H47O2 | 523.3576 | 1.2 | 1.34E+04 | [M+H]+ |
| Glu+Fa13 | 359.2421 | C19H35O6 | 359.2434 | -1.3 | 2.49E+04 | [M+H]+ |
| Glu+Fa13 | 341.2322 | C19H33O5 | 341.2328 | -0.6 | 2.31E+05 | [M+H]+ |
| ketoring | 203.1427 | C14H19O | 203.1436 | -0.9 | 2.53E+05 | [M+H]+ |
|  |  |  |  |  |  |  |

Selected fragmentation data for *Rhodothermus marinus* strain 4252^T^. The highest intensity peaks (seen the figures above) were fragmented and these could be detected in all carotenoid producing strains but not for SB-71.

Target Precursor Ion

Target Precursor ion

Carotenoid Fragment ion containing the isoprene backbone of the carotenoid

Glu+Fa13 Fragment ion containing the glucose and fatty acid

Ketoring Fragment ion containing a keto group on the beta ionene ring
